# Combination Treatment With Intravenous and Oral Calcimimetics for Secondary Hyperparathyroidism in Hemodialysis Patients Who Decline Parathyroidectomy

**DOI:** 10.7759/cureus.81474

**Published:** 2025-03-30

**Authors:** Ryoichi Nakazawa, Akira Onozaki, Kazuhiro Akiyama, Takashi Uchino, Nakanobu Azuma

**Affiliations:** 1 Department of Nephrology, Tokatsu Clinic Hospital, Matsudo, JPN; 2 Department of Surgery, Tokatsu Clinic Hospital, Matsudo, JPN

**Keywords:** calcimimetic, denosumab, hemodialysis, parathyroidectomy, parathyroid hormone

## Abstract

Background and aim: Secondary hyperparathyroidism (SHPT) is a common and serious complication in patients on hemodialysis (HD), leading to significant morbidity and mortality. Parathyroidectomy (PTx) is an established treatment for refractory SHPT, but many patients refuse this surgical option. This study evaluates the efficacy of combination treatment using intravenous and oral calcimimetics in managing SHPT in patients who decline PTx. This study aimed to assess the impact of combination calcimimetic treatment on plasma parathyroid hormone (PTH) levels, mineral metabolism, and clinical outcomes in patients on hemodialysis with SHPT who refuse PTx.

Methods: This retrospective study involved seven patients on HD with refractory SHPT who declined PTX. They were treated with various combinations of intravenous (etelcalcetide or upacicalcet), oral (cinacalcet or evocalcet) calcimimetics, and vitamin D receptor activators (VDRAs), with or without denosumab. Clinical outcomes, including changes in plasma PTH levels, mineral metabolism, and adverse events, were monitored over a period ranging from 10 to 100 months.

Results: Combination treatment significantly reduced plasma PTH levels in all patients (median reduction from 379 pg/mL to 193 pg/mL). No gastrointestinal complications were reported, confirming the tolerability of the regimen. However, two patients developed renal cancer, and one patient died from cardiovascular disease, highlighting the complex comorbidities in this population. These findings underscore the effectiveness of combination calcimimetics in managing SHPT in patients who refuse surgery, although careful monitoring for adverse events is necessary.

Conclusion: The combination of intravenous and oral calcimimetics is an effective therapeutic option for managing SHPT in patients on HD who refuse PTX. While promising, the long-term safety and potential risks of this approach, including the occurrence of malignancies, warrant further investigation in larger prospective studies.

## Introduction

Secondary hyperparathyroidism (SHPT) is a prevalent and severe complication of chronic kidney disease (CKD), particularly among patients undergoing long-term hemodialysis (HD). Persistent elevations in plasma parathyroid hormone (PTH) levels in SHPT disrupt calcium and phosphate homeostasis, leading to derangements in bone remodeling and cardiovascular function. These disturbances substantially increase the risks of fractures, vascular calcification, and cardiovascular morbidity and mortality, thereby exacerbating the already poor outcomes observed in this population [1,2].

Parathyroidectomy (PTx) has historically been considered the gold-standard treatment for severe and refractory SHPT. By surgically reducing parathyroid gland mass, PTx effectively achieves sustained biochemical control. However, the advent of calcimimetic agents, such as cinacalcet, has revolutionized SHPT management by offering a non-surgical alternative. Calcimimetics act on the calcium-sensing receptor (CaSR) of parathyroid cells, suppressing PTH secretion pharmacologically [3,4]. This innovation has significantly decreased the frequency of PTx procedures worldwide.

Despite these advancements, managing SHPT in patients who decline PTx remains a significant challenge. Reasons for refusing surgery may include personal preference, perceived risks, or comorbid conditions precluding safe surgical intervention. While monotherapy with either oral or intravenous calcimimetics has shown efficacy in moderate cases, it is often inadequate for achieving optimal biochemical targets in severe or refractory SHPT [5,6]. However, the use of two calcimimetics may not be allowed by insurance.

Combination therapy involving both intravenous and oral calcimimetics has emerged as a promising strategy to address these therapeutic limitations. This approach leverages the complementary pharmacokinetic and pharmacodynamic properties of different calcimimetic formulations to enhance PTH suppression and mineral metabolism control. However, evidence regarding the clinical efficacy, safety, and long-term outcomes of combination calcimimetic therapy remains sparse, particularly in patients who refuse PTx.

This retrospective study aimed to assess the impact of combination treatment using intravenous and oral calcimimetics on PTH levels, mineral metabolism, and clinical outcomes in patients on HD with refractory SHPT who decline PTx. By addressing this critical gap in the literature, the findings seek to inform personalized management strategies and improve care for this challenging patient population.

## Materials and methods

Study design and patient selection

This retrospective clinical study was conducted as part of a routine clinical audit at the hemodialysis center of Tokatsu Clinic Hospital and its affiliated clinics in Matsudo, Japan. The study focused on patients undergoing HD who received calcimimetics, vitamin D receptor activators (VDRAs), and denosumab as part of their treatment for SHPT. The study design adhered to institutional data-handling protocols and was exempt from institutional review board approvals, as it used aggregated, de-identified patient data. Patients included in the study had refractory SHPT and were managed with calcimimetics, VDRAs, and denosumab between November 2015 and March 2024. Seven patients who declined PTx despite clinical indications were selected for analysis. PTx was recommended in cases where the intact PTH levels exceeded 500 pg/mL, plasma PTH levels were unresponsive to maximal doses of calcimimetics and VDRAs, or hyperphosphatemia or hypercalcemia could not be controlled medically [7]. This study was conducted according to the principles outlined in the Declaration of Helsinki. The Ethics Committee of the Tokatsu Clinic Hospital granted ethical approval (approval number 2024-4).

Data collection

Data were retrospectively extracted from electronic medical records as part of a one-time audit. Demographic information, health-related variables, and treatment regimens were collected using a clinical dashboard. The data included starting and maximum doses of calcimimetics, duration of treatment on HD, plasma intact PTH (iPTH) levels (measured biweekly), serum corrected calcium (calcium concentration {mg/dL} + 4-serum albumin {g/dL}), phosphorus, and alkaline phosphatase levels, dialysate calcium concentrations, and concomitant medications and reasons for treatment discontinuation. Laboratory analyses were performed using standard methods at the Department of Laboratory, Tokatsu Clinic Hospital. Plasma iPTH levels were measured using the Abbott ARCHITECT i2000SR PLUS analyzer with an electrochemiluminescence-based method, while calcium, phosphorus, and alkaline phosphatase were determined using a HITACHI LABOSPECT008a analyzer. Target ranges for serum phosphorus, corrected calcium, and iPTH levels were based on the Japanese Society for Dialysis Therapy guidelines as follows: 3.5-6.0 mg/dL, 8.4-10.0 mg/dL, and 60-240 pg/mL, respectively [7]. Once a year, parathyroid gland size was measured by neck ultrasound using the ellipsoid formula (π/6 × a × b × c, where a, b, and c represent the diameters of the gland in three dimensions) with a Canon Aplio flex ultrasound system [8].

Treatment protocol

Initial treatment involved a single calcimimetic at the maximum dose allowed by Japanese insurance, combined with a VDRA. When PTH control remained inadequate and PTx declined, alternative calcimimetic regimens or denosumab were introduced. Among the seven cases, one patient received a combination of etelcalcetide, VDRA, and denosumab. Four patients were treated with a regimen of etelcalcetide, evocalcet, and VDRA. One patient received etelcalcetide, cinacalcet, and VDRA. One patient was managed with upacicalcet, evocalcet, and VDRA. All patients received dialysis with a dialysate calcium concentration of 2.75 mEq/L to manage calcium-phosphorus balance effectively.

Outcomes measures

The primary outcomes included changes in plasma PTH levels, serum calcium, and phosphorus concentrations. Secondary outcomes included adverse effects such as gastrointestinal intolerance or other clinically significant events. Treatment efficacy and tolerability were assessed using routine laboratory and clinical evaluations.

Statistical analysis

Continuous variables were compared using the Wilcoxon signed-rank test in EZR (Easy R). A p-value <0.05 was considered statistically significant. Data analyses were performed using standard statistical software.

## Results

Patient characteristics

This retrospective study analyzed seven patients on HD with confirmed indications for PTx who declined surgery. The study spanned from November 2015 to March 2024 and included five men and two women aged 52-69 years. The duration of HD ranged from 7 to 36 years (Table 1). Six patients presented with hypercalcemia (serum calcium >10.0 mg/dL), and plasma iPTH levels ranged from 212 to 612 pg/mL despite treatment with calcimimetics and VDRAs (Table 2). High-resolution ultrasonography revealed parathyroid gland volumes ranging from 335 to 3,069 mm^3^, with gland counts varying from two (with autografts in the forearm) to five (Table 1). No fractures were observed during the study period.

**Table 1:** Clinical characteristics of seven patients on hemodialysis who underwent combination treatment. Reduction in iPTH levels (mean±SD) before vs. after combined treatment (p=0.022). AKI: acute kidney injury; ALP: alkaline phosphatase; CGN: chronic glomerulonephritis; F: female; HD: hemodialysis; iPTH: intact parathyroid hormone; M: male; NS: nephrosclerosis

Patient number	Age (years)/ sex	Cause of renal failure	HD vintage (years)	Corrected serum calcium level (mg/dL)	Serum phosphorus level (mg/dL)	Serum ALP level (IU/mL)	Parathyroid gland volume (mm^3^)	Number of parathyroid glands	iPTH (before the treatment) (pg/mL)	iPTH (after the treatment) (pg/mL)
1	54/M	CGN	36	10.2	5.3	106	391	2+autograft	319	282
2	63/F	AKI	12	10.4	7.2	53	419	3	319	285
3	59/M	CGN	16	10.5	3.4	68	671	4	389	126
4	69/M	CGN	25	9.8	6.4	42	335	5	222	142
5	58/M	CGN	8	10.1	6.0	69	543	3	212	175
6	52/M	CGN	28	10.7	6.6	414	3,069	4	583	212
7	67/F	NS	7	10.3	5.8	155	2,068	5	612	126
Mean±SD	60±6	-	19±11	10.3±0.3	5.8±1.2	130±131	1,071±1,069	3.7±1.1	379±161	193±69

**Table 2:** Other characteristics of seven patients who underwent treatment with double calcimimetics. Maximum permitted usage by insurance is as follows: etelcalcetide=15×3/week, IV; upacicalcet=300×3/week, IV; evocalcet= 12/day, p.o.; cinacalcet=100/day, p.o.; maxacalcitol=20×3/week, iv; denosumab=60 mg/6 months, sc. AT: autotransplantation; d: day; IV: intravenous injection; mos: months; Nx: nephrectomy; PTx: parathyroidectomy; p.o.: per os; sc: subcutaneous injection This table summarizes the other characteristics of the seven patients, their treatments, prognosis, and complications, along with the corresponding treatment dosages and administration schedules.

Patient number	Etelcalcetide (mg)	Upacicalcet (µg)	Evocalcet (mg)	Cinacalcet (mg)	Maxacalcitol (µg)	Denosumab (mg)	Other treatment	Prognosis	Complications	Period (mos)
1	15×3	-	7	-	2.5×3	-	PTx+AT	Death	-	13
2	-	300×3	6	-	2.5×3	-	-	-	-	16
3	15×3	-	4	-	2.5×1	×3	-	-	-	24
4	15×3	-	-	50	2.5×3	-	-	-	-	15
5	15×3	-	2	-	2.5×3	-	-	-	-	10
6	15×3	-	-	12.5	2.5×3	×16	-	-	Left renal cancer (Nx)	100
7	10×3	-	12	-	2.5×3	-	-	-	Right renal cancer (Nx)	54

Reduction of plasma PTH levels

**Figure 1:**
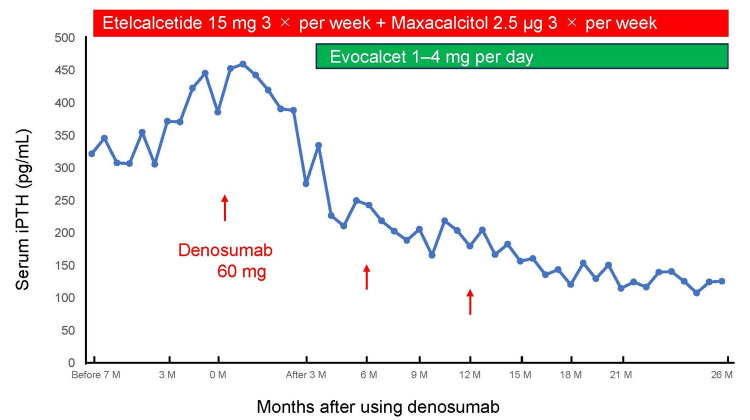
Changes in patient 3's plasma iPTH levels during combination treatment. iPTH: plasma intact PTH

**Figure 2:**
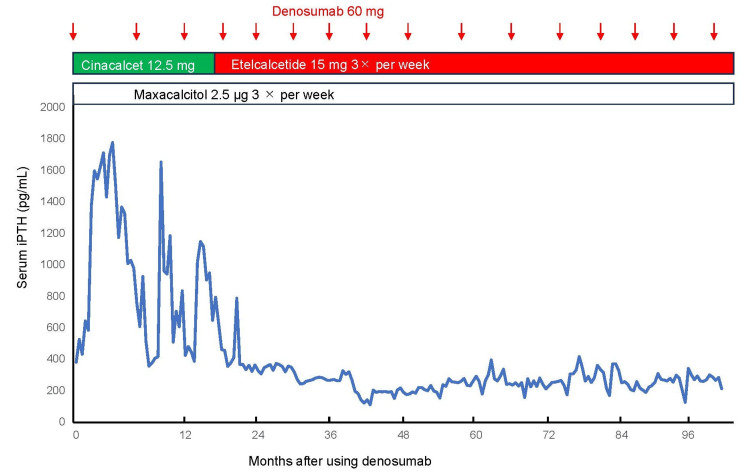
Changes in patient 6's plasma iPTH levels during combination treatment. iPTH: plasma intact PTH

Combination treatment regimens led to a significant reduction in plasma PTH levels across all patients. Detailed changes in PTH levels are presented in Table 1, with trends illustrated in Figures 1, 2. Median PTH levels decreased from 379 pg/mL to 193 pg/mL after initiating combination therapy, demonstrating its efficacy in managing severe SHPT in patients who declined PTx. The consistency of the observed reduction across different treatment combinations highlights the therapeutic potential of this approach.

Tolerability

The combined calcimimetic regimens were well-tolerated, with no gastrointestinal complications reported during the observation period. This supports the feasibility of these regimens, especially for patients with known sensitivities to conventional calcimimetic monotherapy. 

Adverse events

Although the treatment effectively controlled PTH levels, two significant adverse events were noted as follows: (1) renal cancer - two patients were diagnosed with renal cancer by annual abdominal echography, CT imaging, and hematuria, which was confirmed to be derived from acquired cystic disease of the kidney with unilateral removal of the kidney. (2) Cardiovascular mortality - one patient, who had been on dialysis for 36 years, succumbed to cardiovascular disease, a leading cause of mortality among the dialysis population. These findings underscore the complexity of managing SHPT in patients with advanced CKD and highlight the interplay between SHPT, its treatment, and the comorbidities inherent to this population.

Clinical implications

The study findings emphasize the necessity of combination calcimimetic therapy for achieving biochemical control of SHPT in patients who refuse PTx. However, the observed risks associated with long-term treatment underscore the importance of vigilant monitoring and individualized care to mitigate potential adverse effects.

## Discussion

This study highlights two critical aspects of managing SHPT in patients on HD who refuse PTx. First, the results demonstrate that combination treatment with intravenous and oral calcimimetics, targeting distinct domains of the CaSR, effectively reduces plasma PTH levels in this challenging patient population. Second, the unexpected diagnosis of renal cancer in two patients during the study period underscores the complexity of long-term SHPT management and necessitates further investigation into its etiology and implications.

Since the advent of calcimimetics, the therapeutic paradigm for SHPT has shifted substantially from surgical to medical management. This shift is particularly evident in Japan, where the annual number of PTx procedures for SHPT decreased from 1,771 in 2007 to just 67 in 2022 [9,10]. The availability of multiple calcimimetic agents - cinacalcet, etelcalcetide, evocalcet, and upacicalcet - has broadened the range of treatment options. However, patient reluctance to undergo PTx, often due to concerns about surgical risks and personal preferences, has amplified reliance on pharmacological interventions.

The findings of this study align with in vitro evidence suggesting enhanced CaSR activation when intravenous and oral calcimimetics are combined. For instance, the concurrent use of etelcalcetide and cinacalcet has been shown to produce significantly higher intracellular inositol-1-phosphate levels in HEK-293T cells than either agent alone [11-13]. This pharmacological synergy likely underpins the substantial PTH reductions observed in our cohort. Despite these promising results, combination treatments' long-term safety and efficacy remain uncertain and warrant further investigation, as current evidence is predominantly limited to small-scale or preclinical studies.

The diagnosis of renal cancer in two patients during the study period raises pertinent questions about the potential relationship between SHPT, its treatments, and malignancy risk. Patients on HD are at an elevated risk of renal cancer, with a standardized incidence ratio of 4.03 compared to the general population [14,15]. While the causal link between SHPT treatments and cancer remains speculative, this finding underscores the importance of vigilant monitoring and necessitates additional research to elucidate potential mechanisms.

Strict PTH control is central to SHPT management. Recent studies suggest that achieving plasma iPTH levels below 60 pg/mL is not associated with increased mortality, supporting the strategy of aggressive PTH suppression through pharmacological or surgical means [16,17]. However, the benefits of such approaches must be carefully balanced against the potential risks, including possible malignancy development.

This study's limitations include its small sample size, retrospective design, and relatively short follow-up period. These factors constrain the generalizability of the findings and preclude definitive conclusions regarding causality or broader clinical implications. Prospective, large-scale studies are needed to validate the efficacy and safety of combination calcimimetic regimens and to investigate the potential links between SHPT treatments and long-term outcomes, including cancer risk.

## Conclusions

Managing SHPT in patients on HD who decline PTx requires intricate and individualized pharmacotherapy strategies. This study demonstrates that combining dual calcimimetics with a VDRA can effectively control plasma PTH levels in this complex patient population. However, the occurrence of adverse events, such as renal cancer, highlights the need for cautious application of these therapies and further exploration of their long-term safety and clinical implications. Comprehensive, prospective research is essential to establish optimal treatment protocols for SHPT in patients unwilling or unable to undergo PTx.
